# The cyclin-like protein Spy1/RINGO promotes mammary transformation and is elevated in human breast cancer

**DOI:** 10.1186/1471-2407-12-45

**Published:** 2012-01-26

**Authors:** Mohammad Al Sorkhy, Rosa-Maria Ferraiuolo, Espanta Jalili, Agnes Malysa, Andreea R Fratiloiu, Bonnie F Sloane, Lisa A Porter

**Affiliations:** 1 Faculty of Pharmacy, Al Zyatoona Private University, Amman, Jordan; 2 Department of Biological Sciences, University of Windsor, ON N9B 3P4 Windsor, Canada; 3 Department of Biochemistry, University of Calgary, AB T2N 1 N4 Alberta, Canada; 4 Department of Pharmacology, Wayne State University School of Medicine, Detroit, MI 48201, USA

## Abstract

**Background:**

Spy1 is a novel 'cyclin-like' activator of the G1/S transition capable of enhancing cell proliferation as well as inhibiting apoptosis. Spy1 protein levels are tightly regulated during normal mammary development and forced overexpression in mammary mouse models accelerates mammary tumorigenesis.

**Methods:**

Using human tissue samples, cell culture models and in vivo analysis we study the implications of Spy1 as a mediator of mammary transformation and breast cancer proliferation.

**Results:**

We demonstrate that this protein can facilitate transformation in a manner dependent upon the activation of the G2/M Cdk, Cdk1, and the subsequent inhibition of the anti-apoptotic regulator FOXO1. Importantly, we show for the first time that enhanced levels of Spy1 protein are found in a large number of human breast cancers and that knockdown of Spy1 impairs breast cancer cell proliferation.

**Conclusions:**

Collectively, this work supports that Spy1 is a unique activator of Cdk1 in breast cancer cells and may represent a valuable drug target and/or a prognostic marker for subsets of breast cancers.

## Background

The Speedy/RINGO family of proteins are novel regulators of the cell cycle, capable of activating the cyclin-dependent kinases (Cdks) independent of cyclin binding and phosphorylation within the Cdk T-loop [1]. The human Speedy/RINGO homolog, herein referred to as Spy1, is constitutively expressed in most human tissues and is essential for somatic cell cycle progression [2]. Ectopic expression of Spy1 promotes rapid cell cycle progression through G_1_/S phase that is attributed, at least in part, to the activation of Cdk2 [2]. Spy1 can prevent the inhibitory effects of the tumor suppressor p27^Kip1 ^on Cdk2 by directly promoting p27 degradation, suggesting yet another mechanism by which Spy1 can enhance both normal and aberrant cell growth [3-5]. SAGE analysis has shown that Spy1 is expressed at elevated levels in one case of invasive ductal carcinoma of the breast [6]. Spy1 protein levels have also been implicated as a prognostic marker in hepatic carcinogenesis and ectopic overexpression of Spy1 can accelerate mammary tumorigenesis in vivo [6-8]. Of potential importance, two independent linkage studies have resolved that the chromosomal loci at the precise location of Spy1 (2p23.2) may be a candidate site contributing toward breast cancer risk, particularly in women under the age of 50 [9,10]. Hence, how Spy1 levels contribute to the initiation and/or progression of tumorigenesis is of high priority for understanding both normal and abnormal cell growth programs.

Spy1 protein levels are tightly regulated during the cell cycle, being transcriptionally upregulated by the oncogene c-Myc [8,11,12]. We have resolved three residues within the N-terminal region of the protein; T15, S22, and T33 which are essential for targeting Spy1 for ubiquitin-mediated degradation in G2/M phase of the cell cycle [11]. Mutation of these residues generates a non-degradable form of Spy1 (Spy1-TST) which significantly enhances cell proliferation over that of wild-type Spy1 [11]. Herein, we demonstrate that: (1) elevated levels of Spy1 is a transforming event, (2) Spy1-mediated transformation relies on the activation of Cdk1 and may mediate an inhibition of the pro-apoptotic regulator FOXO1, (3) levels of Spy1 protein are highly elevated in aggressive human breast cancers and (4) downregulation of Spy1 can significantly inhibit breast cancer cell growth. Collectively these data support that Cdk1 kinase activity is essential for Spy1-mediated transformation and may indicate a therapeutically relevant mechanism of treating tumors with elevated levels of Spy1 protein.

## Methods

### Cell Culture

The mouse embryonic fibroblast cell line NIH3T3 (ATCC), human embryonic kidney cell line HEK-293 (293; ATCC) and human breast cancer line MDA-MB-231 (MDA-231; ATCC) were maintained in DMEM medium (Sigma) supplemented with 10% (vol/vol) calf serum (Sigma) for NIH3T3 cells, and fetal bovine serum (FBS; Sigma) for 293 cells. The BALB/c mouse mammary epithelial cell line HC11 (Dr. C. Shermanko) and breast cancer MCF7 cells (Dr. T. Seagroves) were maintained in RPMI 1640 medium (Sigma) containing 10% (vol/vol) fetal calf serum and supplemented with 5 μg/ml insulin (Sigma), and 10 ng/ml EGF (Gibco). Human breast MCF10A series cell lines (ATCC & Drs. B. Sloane and F. Miller) were maintained in DMEM-F12 media containing 0.5 ug/ml hydrocortisone, 10 ug/ml insulin, 20 ng/ml human EGF and 5% (vol/vol) horse serum heat inactivated. The MMTV-Myc cell line was derived from a freshly dissected mammary adenocarcinoma from a 3 month multiparous MMTV-Myc female mouse. All cell lines were maintained in a media containing 2 mM L-glutamine (G7513; Sigma), penicillin and streptomycin (15140; GIBCO), and were cultured in a 5% CO_2 _environment. Cells not received from ATCC were tested for tissue/species specific genes and characteristic receptor status via Q-RT-PCR. These tests, as well as testing for mycoplasma, bacteria, fungi contamination and cytogenetic characterization are performed on cells obtained from ATCC.

### Plasmid and mutagenesis

Creation of the Myc-Spy1-PCS3 vector was described previously [13]. Spy1-TST was created using QuickChange PCR Multi-Site-Directed Mutagenesis (Stratagene) of Spy1-PCS3 in 3 sequential steps to generate alanine mutations at positions T15, S22 and T33. Successful cloning was determined by DNA sequencing (Robarts Sequencing Facility; UWO). Plasmids for FLAG-FOXO1 (#9036), HA-Cdk1 (#1888), HA-Cdk1-DN (#1889), pLKO-scrambled control (#8453), the luciferase reporter construct 3xIRS (#13511) and lentiviral constructs: pMD.G (#12259), pMDLg/pRE (#12251) and pRSV-Rev (#12253) were all obtained from Addgene. pLKO Spy1 was cloned to express the short hairpin previously demonstrated to specifically knockdown Spy1 in place of the scrambled sequence in pLKO above [2]. pSUPER (Oligoengine) containing a scrambled siRNA and pSUPER with siSpy1 are previously described [11]. Generous gifts; FOXO1-A3/S249A (Dr. H. Huang; University of Minnesota), luciferase control plasmids (Dr. B. Vogelstein; Johns Hopkins University) and Ras-V12 (Dr. S. Lowe; Cold Spring Harbor).

### Antibodies

Primary antibodies were as follows: Spy1 (NB100-2521; Novus), Myc (9E10 and C19; Santa Cruz), HA (Y11 and F7; Santa Cruz), Cdk1 (ab31687; Abcam), TOTO-3 (T-3600; Molecular Probes), IgG (SC66186; Santa Cruz), Cdk2 (SC-6428; Santa Cruz), Flag (F3040; Sigma) and actin (MAB1501R; Chemicon).

### Transfection/infection methods

Cells were transfected using polyethylenimine (PEI) branched reagent (408727; Sigma). In brief, for experiments using a fixed amount of DNA per construct 10 μg of DNA was mixed with 50 μL of 150 mM NaCl and 3 μL of 10 mg/ml PEI for 10 min then added to a 10 cm tissue culture plate. For transfections requiring more DNA as specified, the relative amounts of NaCl and PEI were scaled up accordingly. Media was changed after 8 h for NIH3T3s and MCF7s and remained overnight for the 293 cells. For viral preparation: media was changed after 8 h transfection of packaging lines and virus harvested 24 h later. Virus was sterilized using a 0.45 μm filter and concentrated using ultracentrifugation for 3 h at 25 K rpm at 4°C. Viral titre was determined using HC11 cells. Using a titre of 10^7^/ml and an MOI of 10, MDA-231 cells were infected ~70-80% confluency in serum free and antibiotic free media containing polybrene (25 μg/mL). Media was replaced 6 h post-infection. Proliferation assays were conducted by counting live and dead cell populations using trypan blue exclusion and using a haemocytometer; counts were also verified using a TC10 automated cell counter (Biorad).

### Cell synchronization and flow cytometry

Cells were synchronized using double thymidine block; cells were cultured in a media containing 2 mM thymidine for 16 h, released to normal media for 8 h, followed by a 14 h block in 2 mM thymidine and then released in 70 ng nocodazole. NIH3T3 cells were synchronized by being cultured in a 2% serum containing media for 24 h, followed by release in standard culture media.

Flow cytometry analysis; cells were carefully collected at indicated times (taking care to include even floating cell populations), washed twice in PBS, and then either used immediately or fixed for future analysis. Fixed cells were resuspended at 2 × 10^6 ^cells in 1 ml of PBS, fixed by the dropwise addition of an equal amount of ethanol, and frozen at -80°C. Within 1 week, fixed cells were pelleted, washed, and resuspended in 300 μl of PBS. Samples of resuspended fixed cells or fresh cells were treated with 1 μl of 10 mg/ml stock of DNase free RNase (Sigma) and 50 μl of 500 mg/ml propidium iodide solution. Cells shown were first gated for size using forward scatter and side scatter parameters. A minimum of 300,000 cells were analyzed per treatment using a Beckman Coulter FC500.

### Immunoblotting (IB) and immunoprecipitation (IP)

Cells were lysed in 0.1% NP-40 lysis buffer (5 ml 10% NP-40, 10 ml 1 M Tris pH 7.5, 5 ml 0.5 M EDTA, 10 ml 5 M NaCl up to 500 ml RO water) containing protease inhibitors (10 μg/ml PMSF, 60 μg/ml aprotinin, 10 μg/ml leupeptin) for 30 min on ice. Bradford Reagent was used to determine the protein concentration (Sigma). 20-30 μg protein were subjected to electrophoresis on denaturing SDS polyacrylamide gels and transferred to PVDF-Plus 0.45U transfer membranes (Osmonics Inc.) for 2 h at 30 V using a wet transfer method. Blots were blocked for 2 h in TBST containing 3% non-fat dry milk (blocker) at RT, primary antibodies were reconstituted in blocker and incubated overnight at 4°C, secondary antibodies were used at a 1:10,000 dilution in blocker for 1 h at RT. Blots were washed three times with TBST following incubation with both the primary and secondary antibodies. Washes were 6 min each following the primary antibody and 10 min each following the secondary antibody. Blots were visualized using Chemilumiminescent Peroxidase Substrate (Pierce) and quantified on an Alpha Innotech HD2 (Fisher) using AlphaEase FC software.

For IP, equal amounts of protein were incubated with primary antisera as indicated overnight at 4°C, followed by the addition of 10 ul protein A-Sepharose (Sigma) and incubated at 4°C with gentle rotation for an additional 2 h. These complexes were then washed 3× with 0.1% NP-40 lysis buffer and resolved by 10% SDS-PAGE.

### Tissue microarray (TMA) analysis

Paraffin embedded TMA slides (cat # BR721 and BR962; US Biomax) consisting of 165 tissue cores in total were deparaffinized and rehydrated in decreasing percentages of ethanol according to the manufacturer's instructions. Antigen retrieval was performed at 95°C in 0.01 M sodium citrate buffer, pH 6.0. Slides were washed in 1× PBS, permeabilized in 1 × PBS/0.2% Triton X-100 at RT followed by 3 washes with 1 × PBS. Sections were blocked in goat serum for 1 h at 37'C, followed by incubation in primary antibody for 2 h at 37°C. Slides were washed twice for 10 min in PBS-1% normal goat serum. Secondary antibodies were applied for 1 h at 37°C, washed 3× for 10 min each in 1 × PBS and incubated 30 min with a nuclear counterstain for TOTO-3. The fluorescent signal was detected and quantified by ScanArray Express (Perkin Elmer Inc.). The Spy1 signal intensity was normalized to nuclear stain signal.

### Soft agar assay

Two layers of agar/media mixture were plated into 60 mm culture dishes. Briefly, 0.6 g of Noble agar (UBS) was suspended in 100 ml DI water to yield a 0.6% bottom agar solution. This mixture was poured into two 50 ml tubes, sealed and placed at 40°C for 40 min. Simultaneously; DMEM media enriched with 20% FBS was also incubated at 40°C. The bottom agar layer contained 1.5 ml of 0.6% agar solution and 1.5 ml of media solution was poured and plated. When solidified, a top agar layer was prepared containing a 3% agar solution. Cells (~2.5 × 10^5^) were harvested, counted and added to the top agar + media mix and then promptly layered onto the bottom agar layer. Plates were set for 1 h under sterile conditions and then incubated at 37°C in 5% CO_2 _for 12-14 days. Triplicate transfections for each experiment were observed using light microscopy and pictures were taken using an Apha Innotech HD2 camera. Colonies were counted manually using light microscopy.

### Focus assay

Low passage cells were grown in 10 cm plates, transfected as above and grown to confluence. Media with 2% calf serum was gently changed every other day for up to two weeks. Plates were stained with 0.5% crystal violet, photographed using an Apha Innotech HD2 camera and the number of colonies were counted manually.

### Fat pad transplants

Mice were maintained following the guidelines of the Canadian Council on Animal Care under the ethical approval of the Animal Care Committee, University of Windsor (AUPP #06-19). Fat pad transplant assays were conducted using BALB/c mice, which are syngenic for the HC11 cell line as previously described [8]. In brief, 5 × 10^5 ^cells were injected into the cleared fat pad of 4th inguinal mammary glands of 22 day old mice and allowed to grow for 1 to 8 week. Tumor presence was monitored weekly by palpitation of the gland. Animals were sacrificed humanely at the specified time points and glands dissected for analysis. Tumor volume was calculated as length (mm) × width (mm) × height (mm) using manual calipers.

### Kinase assays

Cells were transfected, cultured in 10% FBS and lysed in 0.1% NP-40 lysis buffer. 16 h post-transfection IP was carried out as described above and precipitates were washed four times prior to the addition of 50 μl of kinase buffer (50 mM Tris-HCl pH 7.5, 10 mM MgCl_2_, 1 mM DTT, 20 mM EGTA, 50 mM ATP, 10 μCi of [γ-^32^P]ATP) and 74 μg/ml H1 histone (Boehringer Mannheim). Reactions were incubated for 10 min at 30°C, sample buffer was added to stop the reaction and 50 μl of each sample were analyzed by 10% SDS-PAGE. Incorporated phosphorylation was visualized using a Cyclone storage phosphor system and quantified using OptiQuant software (Perkin Elmer). IPs were subsequently probed on the same membrane.

### Luciferase assay

Cells were transfected with the appropriate luciferase reporter construct, harvested 24 h post-transfection and 50 ul of cell suspension was mixed with 50 ul of Bright-glo reagent (E2620; Promega). Luminescence spectra of the samples were measured using a plate reader (Wallac Victor 1420).

### Statistical analysis

Results are presented as the mean ± standard error. Statistical significance was assessed through either a student's *t*-test (analysis of two means) or analysis of variance (ANOVA) followed by a post-hoc Tukey test (for more than two means with equal variances) or post-hoc Bonferroni (for more than two means without equal variances). StatSoft's STATISTICA software was used for all analysis.

## Results and Discussion

**Figure 1:**
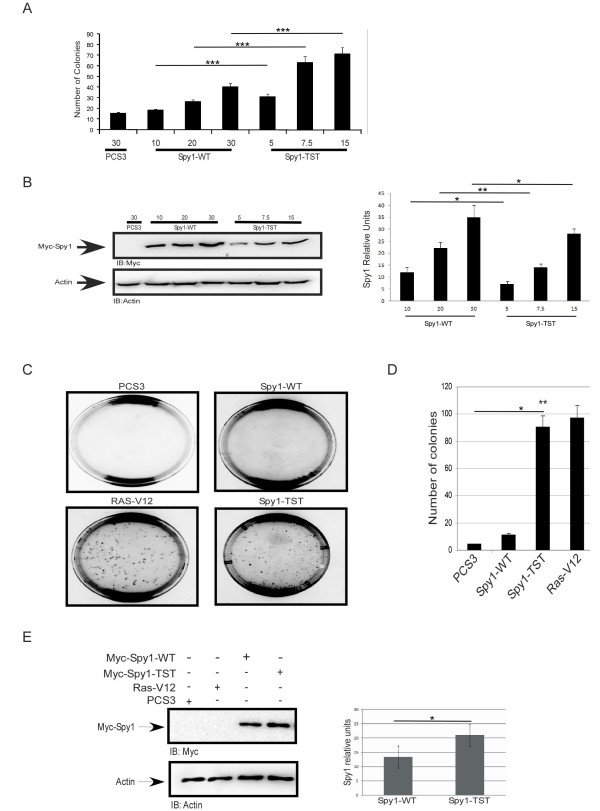
**Spy1 stable protein induces anchorage independent growth**. (A) 293 cells were transfected with different concentrations of Myc-Spy1-WT, Myc-Spy1-TST or 30 μg of empty PCS3 vector control. Soft agar assay was carried out and plates photographed and quantified on day 14. Foci were averaged over 3 separate transfections for each experiment. n = 3 (B) Western blot analysis of one representative experiment; densitometry in lower panel. n = 3 (**C**) Representative foci after 14 day soft agar assay visualized using light microscopy. Ras-V12 is transfected as a positive control. n = 3. (**D**) Total numbers of foci were counted over 3 separate plates using separate transfections for each experiment. n = 3. Error bars reflect SE between triplicate experiments. *t *test was performed;** *P *≤ 0.01. (E) Western blot analysis of experiments in Figure. **1C & D**. Quantified using densitometry followed by normalization for Actin levels. (A-E) Error bars reflect SE between triplicate experiments. *t *test was performed;* *P*≤ 0.05, ** *P *≤ 0.01, *** *P *≤ 0.001.

Spy1 is an essential cell cycle regulator capable of enhancing cell growth and inhibiting apoptosis [2,14]. Spy1 has been implicated as a prognostic marker in hepatocarcinoma, and increased levels accelerate mammary tumour formation in mouse models [7,8]. To further determine whether accumulating levels of Spy1 protein represent a transforming and potentially oncogenic event, HEK 293 cells were transfected with increasing amounts of Spy1-WT or Spy1 with a modified N-terminal degradation signal (Spy1-TST) and soft agar assays were performed. Enhancing or stabilizing Spy1 protein levels resulted in a concentration dependent increase in colony formation (Figure 1A). It was noted that while protein levels and subsequent colony formation increased proportionately with the amount of cDNA transfected, colony formation was not simply a reflection of total amount of Spy1 protein (Figure 1A & B). Using approximately half the amount of transfected DNA for Spy1-TST resulted in a statistically significant reduction of overall protein levels of ~30%, but at least a 2-fold increase in colony formation with high statistical significance (compare 30 ug Spy1-WT vs. 15 ug Spy1-TST). This suggests the possibility that threshold levels or stabilization of the Spy1 protein triggers a unique mechanism that may contribute toward Spy1-mediated tumorigenesis. The significant increase in colonies seen with the Spy1-TST mutant over Spy1-WT supports that the G2/M degradation mechanism previously described [11] may provide a protective barrier against this potentially oncogenic pathway.

To test this hypothesis, soft agar assays were conducted using similar protein levels of either Spy1-WT or Spy1-TST, along with activated Ras (Ras-V12) as a positive control and empty pCS3 as a negative control (Figure 1C). Colonies were formed in the presence of Ras-V12 as well as Spy1-TST, but no colonies were present in the negative control and few colonies were detected for Spy1-WT (Figure 1C). Quantification over three separate experiments demonstrated that Spy1-TST yielded 4 times more colonies than the Spy1-WT counterpart (Figure 1D). Densitometry analysis of protein levels of transfected Spy1 at the time of seeding, normalized using Actin levels, show less than a 2 fold increase in the overall Spy1 protein levels with Spy1-TST (Figure 1E). The modest increase in protein accumulation with Spy1-TST is not surprising given that we are utilizing asynchronous cells and there are multiple mechanisms for regulating Spy1 protein levels [11,12]. Functionally, this supports that the transforming properties of Spy1-TST are not explained merely by the accumulation of overall amounts of protein.

**Figure 2:**
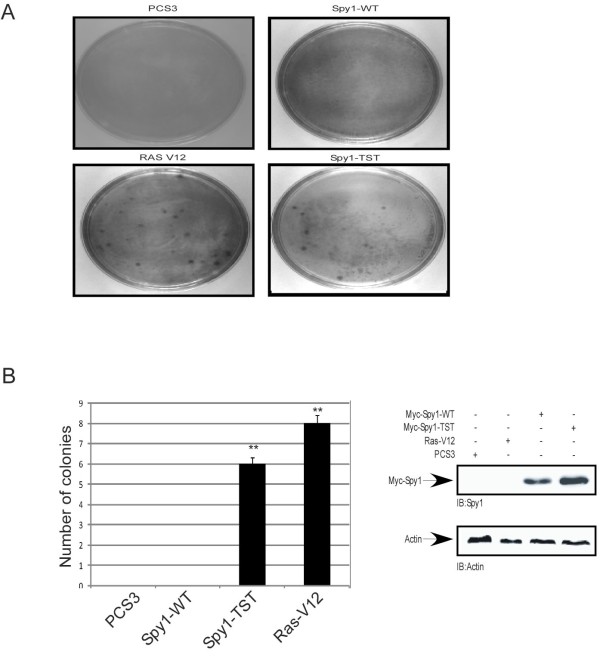
**Elevated Spy1 levels prevent contact inhibition**. (A) Representative views of focus formation assays in NIH3T3 cells transfected with Myc-Spy1-WT, Myc-Spy1-TST, positive control Ras-V12 or empty vector PCS3 control. n = 3 (B) Quantification of the number of colonies. Error bars reflect SE between triplicate experiments. *t *test was performed;** *P *≤ 0.01. n = 3 (right panel) Western blot analysis of one representative lysate from A-B.

To further test this result on the parameters of contact inhibition we performed a foci formation assay using the NIH3T3 cell line (Figure 2A). A significantly higher number of foci were formed from the Spy1-TST and Ras-V12 expressing cells as compared to Spy1-WT counterparts, negligible foci were formed in the control transfected cells (Figure 2B). Modest difference in relative protein levels (Figure 2B, right panel) further support, as above, that Spy1-TST effects on cell growth properties are not adequately explained by increases in overall protein levels.

**Figure 3:**
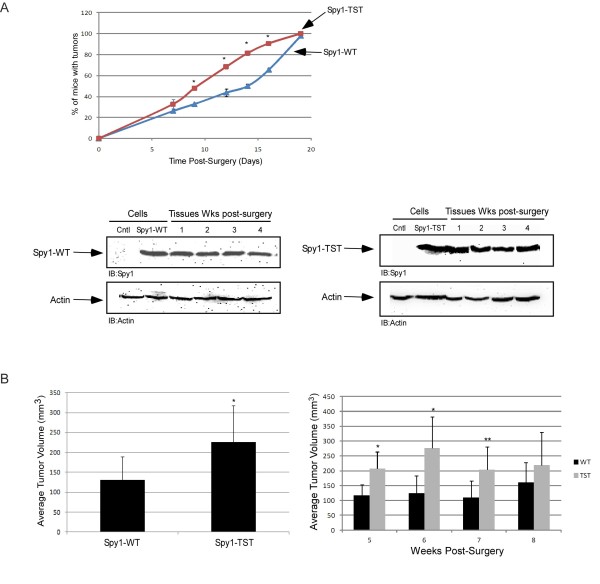
**Spy1 stable protein accelerates tumorigenesis in vivo**.**(A) **Percentage of mice presenting with palpable tumors from 0-19 days post-transplant. Each data point represents 4 mice per indicated construct. The entire experiment was repeated three times using three independently derived overexpressing cell lines for each construct. Mann-Whitney Test was performed (p < 0.05). (A; lower blot) Western blots were conducted to measure the stability of Myc-Spy1-WT (left) and Myc-Spy1-TST (right) from representative time points. Empty vector control (Cntl) cell expression levels of Spy1 are seen in lane 1 of each blot. **(B) **Total tumor volume was calculated for both Spy1-HC11 (Spy1-WT) and Spy1-TST-HC11 (Spy1-TST) transplanted glands. Results were taken from 45 transplants using cells from 3 separate transfections. Error bars reflect SE between transplants from different transfections. Left hand panel reflects overall volume, right hand panel reflects volumes of tissues taken over a month post-transplant.

Spy1 significantly accelerates tumor formation when HC11 cells overexpressing Spy1 were injected into cleared fat pad of BALB/c mice [8]. Mammary fat pad transplants were performed to determine the effect and timing of Spy1-TST in vivo as compared to similar protein levels of the overexpression of Spy1-WT (Figure 3A-B). The fourth inguinal mammary glands of 22 day old BALB/c were cleared and the left fat pad injected with control Spy1-WT-HC11, whereas the right fat pad were injected with Spy1-TST-HC11 cells. One week following transplantation, mice were palpated daily to determine the onset of tumor growth. Tumor onset occurred more rapidly in the Spy1-TST injected glands, with 50% of the mice presenting with tumors by day 8 as compared to day 13 in the Spy1-WT mouse population (Figure 3A). This was statistically significant as assessed by the Mann-Whitney (shown) as well as by Chi-square (p = 0.0003) and the non-parametric Wilcoxin test (p = 0.008). After 5 weeks post-transplantation, mice were sacrificed to determine the extensiveness of tumor growth. While 100% of the glands exhibited invasive tumors at this time, the average total volume of Spy1-TST tumors was found to be almost twice that of the Spy1-WT (Figure 3B). The marked increase in transformation efficiency in vitro and the subtle, albeit significant, effects on tumour growth in vivo of the TST-Spy1 mutant support that overriding the degradation of Spy1 during G2/M may result in the activation of unique pathways that contribute toward Spy1-mediated tumorigenesis. The ultimate formation of tumors in 100% of animals with Spy1-WT (Figure 3A), and the fact that tumour volume differences are most significant at the earliest time points post-surgery (Figure 3B; right panel), support that, as seen in Figure 1A, threshold levels of Spy1 protein may also contribute toward this tumorigenic mechanism.

**Figure 4:**
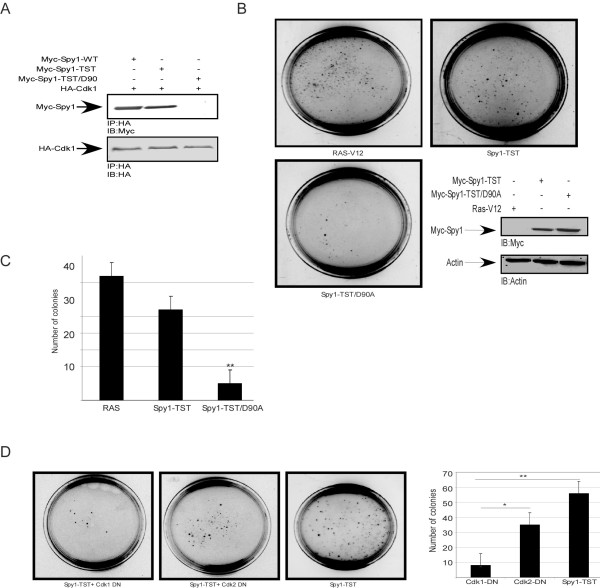
**Spy1 mediated colony formation is Cdk1 dependent**. 293 cells transfected with the indicated constructs were **(A) **immunoprecipitated for HA tagged-Cdk1 and blotted for Myc and HA tag. n = 3 **(B) **imaged after 14 days in a soft agar assay. Transfections and protein levels were monitored by western blot analysis (inset). n = 3 **(C) **Quantification of colony number over three separate transfections. **(D) **Soft agar assays of cells transfected in triplicate with Myc-Spy1-TST in the presence or absence of Cdk dominant negative constructs (Cdk1-DN or Cdk2-DN). n = 3 Plates are imaged and quantified at day 14. Errors bars reflect SD between triplicate experiments. ANOVA with post hoc analysis using Tukey test was performed;* *P *≤ 0.05, ** *P *≤ 0.01.

Spy1 protein is known to regulate cell cycle progression at least in part through the direct binding to Cdks [2]. In somatic cells the primary partner for Spy1, and an essential regulator of Spy1-mediated proliferation, appears to be the G1/S Cdk, Cdk2 [2]. Spy1 is capable of also binding and activating Cdk1 when overexpressed [15]. To determine the role of Cdk proteins on Spy1-mediated transformation we utilized a mutant form of Spy1-TST where the aspartic acid residue at position 90 is mutated to a nonpolar alanine group (Spy1-TST/D90A), a modification previously demonstrated to significantly reduce Spy1-Cdk binding [15]. We demonstrate that this mutation also abrogates the ability of Spy1-TST to interact with Cdk1 (Figure 4A). The Spy1-TST/D90A mutation reduced colony formation in soft agar approximately 5-fold compared to Spy1-TST (Figure 4B & C). To further investigate the relative contribution of each kinase individually to this effect, soft agar assays were performed using Spy1-TST in the presence of the dominant negative form of either Cdk1 (Cdk1-DN) or Cdk2 (Cdk2-DN) (Figure 4D). Interestingly, Cdk1-DN reduced colony formation by ~60% with high statistical significance over 3 separate trials while Cdk2-DN demonstrated reduced colony numbers but this result was not statistically significant. This was not due to inefficient function of the constructs as both DN constructs effectively reduced the kinase activity of their relevant Cdk (Additional file 1: Figure S1). Collectively this data supports that the oncogenic function of Spy1 is dependent, at least in part, on the binding and activation of Cdk1.

Spy1 protein is known to regulate cell cycle progression at least in part through the direct binding to Cdks [2]. In somatic cells the primary partner for Spy1, and an essential regulator of Spy1-mediated proliferation, appears to be the G1/S Cdk, Cdk2 [2]. Spy1 is capable of also binding and activating Cdk1 when overexpressed [15]. To determine the role of Cdk proteins on Spy1-mediated transformation we utilized a mutant form of Spy1-TST where the aspartic acid residue at position 90 is mutated to a nonpolar alanine group (Spy1-TST/D90A), a modification previously demonstrated to significantly reduce Spy1-Cdk binding [15]. We demonstrate that this mutation also abrogates the ability of Spy1-TST to interact with Cdk1 (Figure 4A). The Spy1-TST/D90A mutation reduced colony formation in soft agar approximately 5-fold compared to Spy1-TST (Figure 4B & C). To further investigate the relative contribution of each kinase individually to this effect, soft agar assays were performed using Spy1-TST in the presence of the dominant negative form of either Cdk1 (Cdk1-DN) or Cdk2 (Cdk2-DN) (Figure 4D). Interestingly, Cdk1-DN reduced colony formation by ~60% with high statistical significance over 3 separate trials while Cdk2-DN demonstrated reduced colony numbers but this result was not statistically significant. This was not due to inefficient function of the constructs as both DN constructs effectively reduced the kinase activity of their relevant Cdk (Additional file 1: Figure S1). Collectively this data supports that the oncogenic function of Spy1 is dependent, at least in part, on the binding and activation of Cdk1.

**Figure 5:**
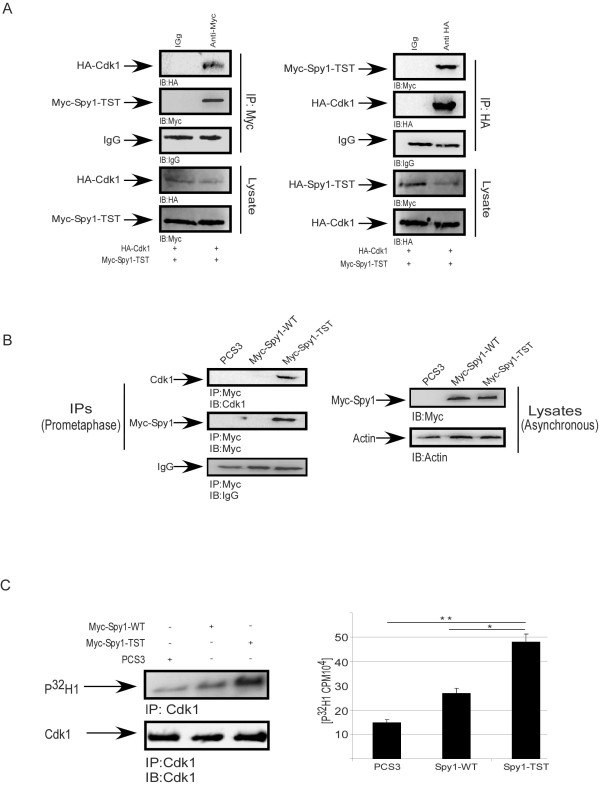
**Spy1 stable protein binds and activates Cdk1**.**(A) **293 cells transfected with HA-Cdk1 and Myc-Spy1-TST were treated with 70 ng Nocodazole for 16 h followed by IP for either Myc or HA. IP with IgG was used as a negative control. Top panels indicate IPs and bottom panels represent the lysate loading controls. n = 2 **(B) **293 cells transfected with the indicated constructs were IPd for Spy1 using the Myc tag and analyzed by 10% SDS-PAGE/IB. n = 3 **(C) **293 cells transfected with the indicated constructs were treated with 70 ng nocodazole for 16 h. Cdk1 IPs were subject to a H1 phosphorylation assay followed by 10% SDS-PAGE. Membranes were imaged on a Cyclone phosphorimager then probed with Cdk1 and imaged on an Alpha Innotech HD2. n = 3 Errors bars reflect SE. *t *test was performed *P < 0.05;**P < 0.01.

We previously demonstrated that Spy1-TST remains stabilized at pro-metaphase of mitosis when microtubule polymerization was prevented using nocodazole treatment; a time point where Spy1-WT was completely degraded [11,12]. Hence, we sought to determine whether aberrant degradation of Spy1 will result in elevated Cdk1 activity during mitosis. Cdk1 has been shown to play an important role in different human cancers, aberrant Cdk1 activation has been described in number of primary tumors [16], providing a potential novel mechanism for Spy1- mediated oncogenic effects. Reciprocal immunoprecipitation of exogenously expressed Spy1-TST (Figure 5A; left panels) or Cdk1 (right panels) demonstrate that these proteins interact in cells blocked with nocodazole. We also demonstrate this interaction using endogenous Cdk1 (Figure 5B). To further investigate if Spy1-TST expression leads to unique activation of Cdk1, cells overexpressing Spy1-WT or Spy1-TST were synchronized in prometaphase followed by immunoprecipitation with Cdk1 antibody and subject to an in vitro kinase assay (Figure 5C). Spy1-TST significantly increased substrate (histone) phosphorylation demonstrating approximately a 3-fold increase over control transfected cells and approximately a 2-fold increase over Spy1-WT transfected cells (Figure 5C). Control transfected cells were collected prior to synchronization to ensure that both Spy1-WT and Spy1-TST were transfected (Figure 5B; right panels). Several reports have shown that aberrant activation and/or elevated levels of Cdk1 or upstream activators are implicated in several forms of human cancer [17-20]. Spy1 binding to its relative Cdk partners has demonstrated unique substrate specificity [21], offering the possibility that Spy1 binding to Cdk1 is uniquely required to promote the oncogenic properties of Cdk1.

**Figure 6:**
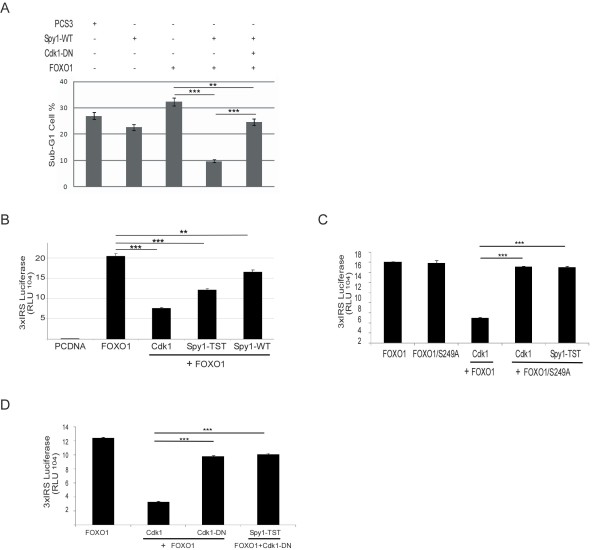
**Spy1 inhibits FOXO1 induced apoptosis through Cdk1 activation**.**(A) **Flow cytometry analysis was conducted using no less than 100,000 transfected 293 cells per data point. Percentage of cells in each phase was determined by CPX analysis. Graphical representation of the overall percent of cells from each treatment harboring a DNA content less than G1 (sub-G1) (flow data presented in Additional file 2: Figure S2A). Error bars reflect SEM between 3 separate transfections. **(B-D) **Luciferase reporter activity for FOXO1 in cells expressing the indicated constructs. **(B) **Errors bars reflect SE. ANOVA with post-hoc Bonferroni correction was performed ** *P *< 0.01, ***P < 0.001. n = 3. **(C-D) **Error bars reflect SE of triplicate transfections within one representative experiment. ***P < 0.001. n = 2.

Huang and colleagues reported that Cdk1 activation inhibits the transcriptional and apoptotic activities of the transcription factor FOXO1, thereby potentiating Ras-mediated oncogenesis [22]. To investigate the effect of Spy1 on FOXO1-induced apoptosis, HEK-293 cells were transfected with FOXO1 in the presence or absence of Spy1-WT or Spy1-TST and/or variants of Cdk1 (Figure 6A-D). Double transfection with FOXO1 and Spy1-WT resulted in significant reduction of a sub-G1 population of cells induced by FOXO1 (Figure 6A). To test if this reduction was mediated through Cdk1 activation, a Cdk1-DN was transfected along with Spy1-WT and FOXO1 (Figure 6A). The effect of Spy1-WT was indeed reversed by the introduction of the Cdk1-DN, supporting that Spy1 inhibition of apoptosis is due to Cdk1 activation. To further investigate the effect of Spy1 on FOXO1 transcriptional activity, a series of luciferase assays were performed (Figure 6B-D). Ectopic expression of Spy1-TST reduced FOXO1 transcriptional activity as significant as the expression of Cdk1 (Figure 6B). It has been demonstrated that Cdk1 phosphorylates FOXO1 on S249, a modification that inhibits FOXO1 transcriptional activity [22,23]. Hence, to determine if Spy1-Cdk1 also functions through this site we utilized a mutant of FOXO1 unable to be phosphorylated by Cdk1; FOXO1-S249A (Figure 6C) [22,23]. Ectopic transfection of Spy1-TST with FOXO1-S249A prevented the inhibition of FOXO1 transcription by Cdk1 and by Spy1-TST. Furthermore, Spy1-TST was unable to suppress FOXO1-mediated transcription in the presence of Cdk1-DN (Figure 6D). These results support that Spy1 is capable of inhibiting FOXO1-mediated transcription through the activation of Cdk1.

**Figure 7:**
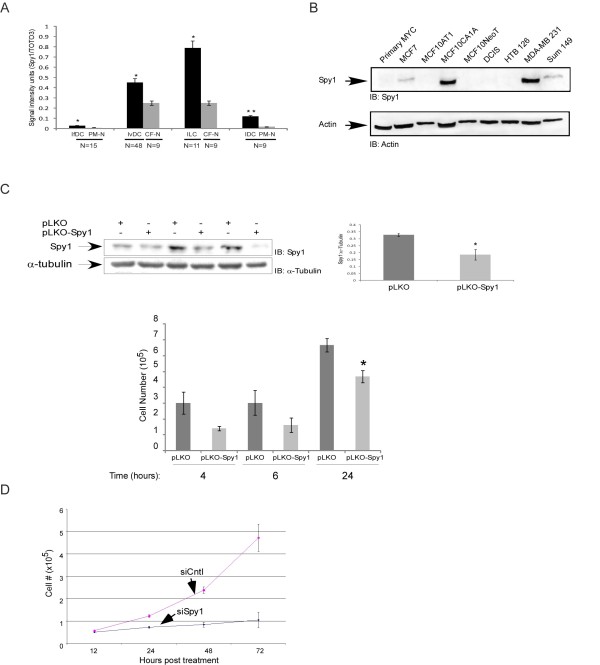
**Spy1 protein levels are elevated in human breast cancers**.**(A) **TMAs containing cores from invasive ductal carcinoma (IvDC), infiltrated ductal carcinoma (IfDC), intraductal carcinoma (IDC) and invasive lobular carcinoma (ILC) as well as pair-matched normal (PM-N) or cancer-free patients (CF-N) were analyzed for Spy1 expression. The Spy1 signal intensity was normalized to nuclear stain (TOTO-3/PI) signal. Patient numbers are indicated below the sample (N). **(B) **Breast normal and cancer cell lines were analyzed by western blot analysis. Actin was used as a loading control. One representative blot of 2. **(C) **Western blot of 3 replicate infections of pLKO control or pLKO Spy1 in MDA-231 cells. Middle panel represents the densitometry values over 3 separate experiments. Where Spy1 is corrected for actin levels. Lower panel reflects cell counts at 4, 6, and 24 h post-infection. (All panels) Data shown is mean ± s.d. Student's *t*-test was performed * *P *< 0.05;**P < 0.01. **(D) **Knockdown effects on cell counts of MCF7 cells over 72 h after transfection with either pSUPER empty vector (siCntl) or pSUPER-Spy1 (siSpy1) over 3 separate transfections. Data shown is mean ± s.d.

Spy1 protein levels are elevated in human hepatocarcinoma and overexpression of Spy1 protein promotes tumorigenesis in mouse models; however, whether Spy1 protein levels are implicated in human breast cancer has not been previously demonstrated [6,7]. TMAs containing a total of 165 cores of the most prevalent forms of breast cancer were used to determine whether levels of Spy1 protein are indeed implicated in human breast cancer. Spy1 antibody specificity was previously published [11] and is further confirmed in Figures 7B and 7C. This data demonstrates that Spy1 is significantly elevated in all forms of breast cancer tested over pair-matched adjacent or normal tissue (Figure 7A). Protein levels of Spy1 were highest in invasive lobular carcinoma and the most significant changes in levels were found in intraductal carcinoma. These data were quantified digitally (Figure 7A) and were confirmed via manual imaging of the TMA slides (Additional file 2: Figure S2B). Spy1 levels have been demonstrated to be high in all proliferative normal mammary tissue [8], we ran a number of breast cancer lines together to select the lines expressing the highest level of Spy1 protein for functional analysis (Figure 7B). Spy1 protein levels were found to be at particularly high levels in the aggressive MDA-231 cell line. Knockdown of Spy1 protein levels in these lines using lentiviral shRNA significantly impaired growth of the by 24 h (Figure 7C). It is notable that although identical numbers of cells were seeded cell numbers at early time points were also lower with Spy1 knockdown, albeit not statistically significant. Cells infected with shSpy1 appear to stop growing after 24 h, hence complicating analysis at later time points but also supporting that Spy1-mediated activity may play a critical role in the growth properties of subsets of breast cancer. To study a longer time course Spy1 was also knocked down with siRNA previously described [11] using the slower growing breast cancer line MCF7, expressing significantly less Spy1 than the MDA-231 cells (Figure 7D). Spy1 knockdown significantly impaired growth for up to 72 h post-infection. Our results support the importance of further examining these effects in vivo. These future experiments are dependent on developing mechanisms of more homogeneous and stable Spy1 knockdown.

## Conclusions

Collectively, this work supports that critical levels of Spy1 protein trigger transformation dependent upon the activation of the G2/M cyclin-dependent kinase, Cdk1. We further show that this mechanism is sensitive to inhibition by the apoptotic regulator FOXO1. We have demonstrated for the first time that levels of Spy1 are elevated in all human breast cancer samples tested and that knockdown of Spy1 can reduce breast cancer cell growth and may represent a novel target for breast cancer therapy.

## Competing interests

The authors declare that they have no competing interests.

## Authors' contributions

MAS carried out the majority of the experiments described herein and aided in the draft of the manuscript. R-MF carried out Figure 3, prepared suppl. Figure 2, ran repeats of several experiments and worked on the manuscript and figures. EJ carried out Figure 7B and provided technical support. AM provided Figure 7Cand statistical analysis. ARF carried out repeats of soft agar assays. BFS is a valuable collaborator, supplying the various breast cancer cell lines and expertise regarding their characteristics. LAP funded the project and had a lead role in study design, interpretation of the data and manuscript preparation. All authors edited the manuscript and provided comments on the intellectual content, and have approved the final manuscript.

## Pre-publication history

The pre-publication history for this paper can be accessed here:

http://www.biomedcentral.com/1471-2407/12/45/prepub

## Supplementary Material

Additional file 1 **Figure S1 Lysates from Figure **4D**were IPd with Cdk1/Cdk2 antibody and subjected to H1 phosphorylation assay followed by SDS PAGE analysis**. One representative experiment of 2. Click here for file

Additional file 2 **Figure S2 (A) Flow profiles from samples described in Figure **6A**are noted**. Transfections are listed above each profile and the relative% of cells falling within the SubG1 population noted on each figure. This is one representative experiment of 3, values noted in 6A are averages over 3 individual experiments. (**B**) Representative TMA samples as depicted by microscopy. Spy1 staining is indicated in green with Alexa-488 secondary. Number on panel indicates the core number. All images are taken at 10×, scale bars indicate 250 uM. Click here for file
