# Supplementary figures and images for: The cyclin-like protein Spy1/RINGO promotes mammary transformation and is elevated in human breast cancer
Source: BMC Cancer. 2012 Jan 26;12:45. doi: 10.1186/1471-2407-12-45 (PMC3294245; doi:10.1186/1471-2407-12-45)

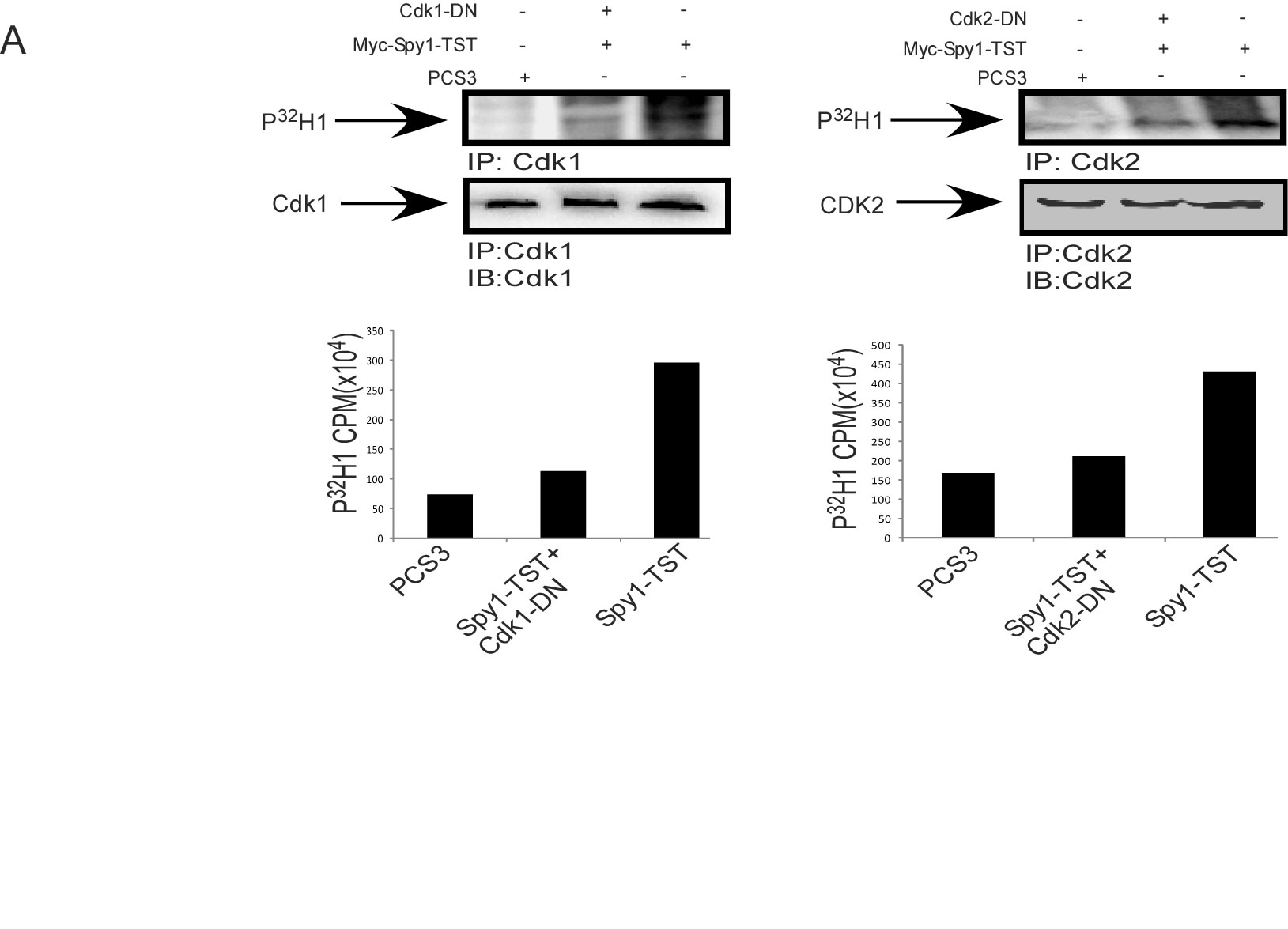

Supplement: Additional file 1 — Figure S1 Lysates from Figure 4Dwere IPd with Cdk1/Cdk2 antibody and subjected to H1 phosphorylation assay followed by SDS PAGE analysis. One representative experiment of 2. [file 1471-2407-12-45-S1.JPEG]

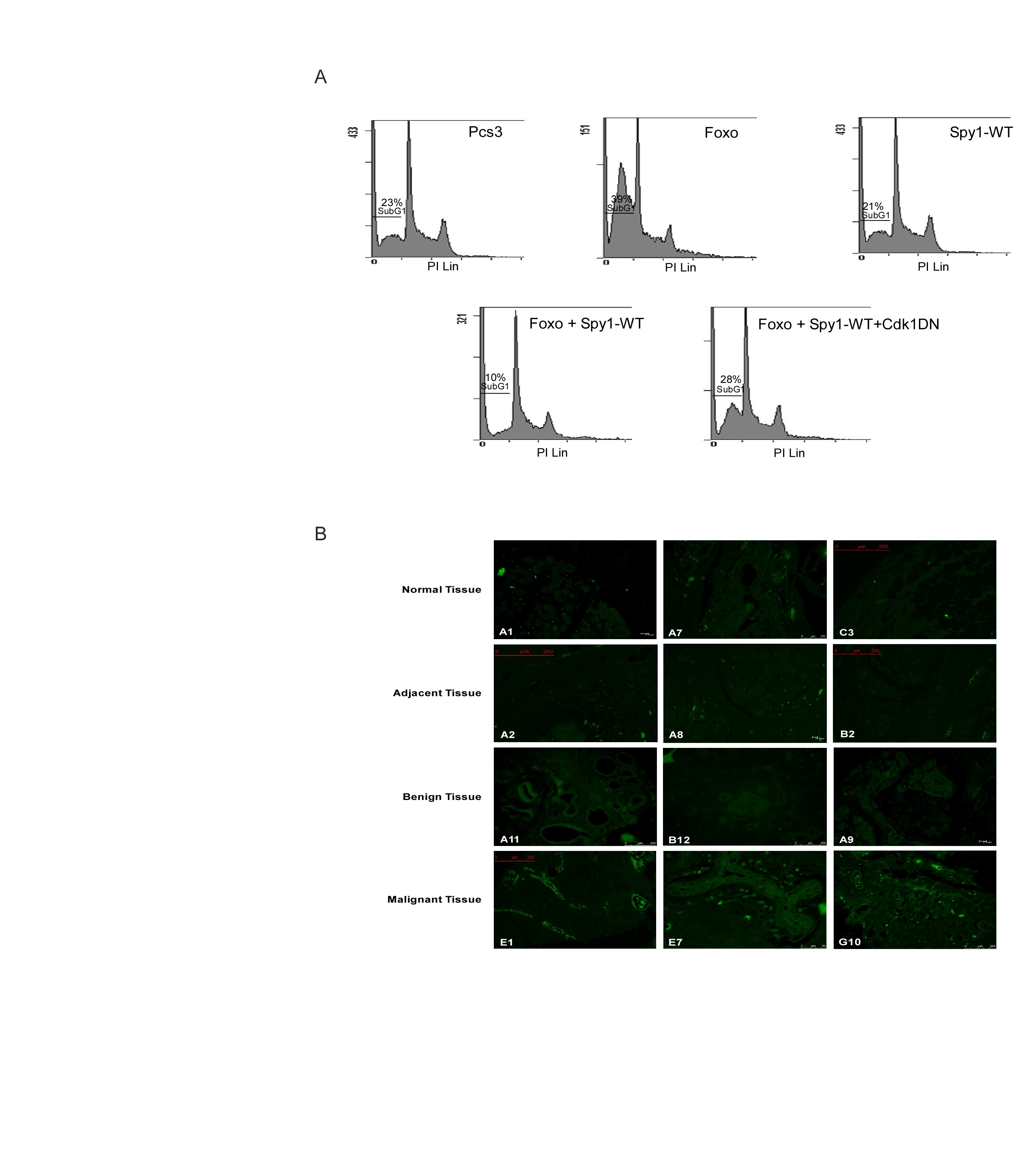

Supplement: Additional file 2 — Figure S2 (A) Flow profiles from samples described in Figure 6Aare noted. Transfections are listed above each profile and the relative% of cells falling within the SubG1 population noted on each figure. This is one representative experiment of 3, values noted in 6A are averages over 3 individual experiments. (B) Representative TMA samples as depicted by microscopy. Spy1 staining is indicated in green with Alexa-488 secondary. Number on panel indicates the core number. All images are taken at 10×, scale bars indicate 250 uM. [file 1471-2407-12-45-S2.JPEG]
